# Motor skills and the relation between preterm birth and working memory during preadolescence

**DOI:** 10.1111/dmcn.70057

**Published:** 2025-10-23

**Authors:** Shu‐Shih Hsieh

**Affiliations:** ^1^ Department of Psychology Kingston University London Kingston upon Thames UK

## Abstract

**This commentary is on the original article by Ludyga et al. on pages** 784‐791 **of this issue.**

Research has shown that children born preterm may exhibit poorer performance in higher‐order cognitive domains, such as executive function. In particular, visuospatial working memory is a critical domain of cognition during childhood, as it may predict future educational and occupational/socioeconomic achievement. Studies have shown that children born preterm tend to have inferior visuospatial working memory performance compared to children born at term.^1^ Additionally, children born preterm may also demonstrate inferior motor skills (e.g. hand‐eye coordination, manual dexterity, balance) relative to their term‐born counterparts. This is a concern given the close developmental link between motor skills and visuospatial working memory capacity.^2^

Under this context, the study by Ludyga et al. provides remarkable insights into the complex relations across preterm birth, visuospatial working memory, and motor skills.^3^ In their case–control matching study, Ludyga et al. demonstrated that very preterm birth (<32 weeks of gestation) is associated with poorer visuospatial working memory performance during preadolescence, and this relation is fully mediated by individual differences in motor skills. Further, using the contralateral delay activity component from electroencephalography (a marker of working memory capacity during encoding and attention filtering), the authors further confirmed the fully mediating role of motor skills in the association between preterm birth and reduced visuospatial working memory capacity. These findings substantiate the role of motor skills in the developmental trajectory of visuospatial working memory in children born preterm.

Despite these valuable insights, several outstanding challenges should be acknowledged. First, the cross‐sectional design limits the ability to explore long‐term trajectories between motor skills and development of visuospatial abilities. Analysing a single time point of data provides limited implications for long‐term adaptations. More longitudinal studies on this topic are necessary. Second, the sample size (*n* = 106) and recruitment strategy (i.e. dichotomize participants by term vs very preterm) limited the possibility to examine whether a linear or curvilinear relation between gestational age and visuospatial working memory ability exists, as well as whether this relation is mediated by motor skills. Studies with larger sample size and employ subgroup analyses involving children with varied gestational ages are necessary to address this challenge. Third, the data analysis did not consider neurodevelopmental conditions, such as attention‐deficit/hyperactivity disorder, developmental coordination disorder, and autism spectrum disorder. Research has shown that children with neurodevelopmental conditions tend to have poorer motor skills and executive function than their neurotypical counterparts.^4^, ^5^ Inclusion or exclusion of these groups of children may have altered the results. Sensitivity analyses that consider the presence of neurodevelopmental conditions could further clarify these complex relations.

Findings from the Ludyga et al. study have important implications for practice and future research. From a practice perspective, interventions targeting motor skill development may be essential in mitigating the adverse effects of preterm birth on executive function (visuospatial working memory in particular) during preadolescence. Their study also opens new avenues for future research. For example, the interrelation between motor skills and visuospatial working memory may be influenced by abnormalities in the encoding and retrieval of information that is not captured by the contralateral delay activity component from electroencephalography. It would also be relevant to refine the mediation model, for instance, by identifying which domains of motor skills may exert stronger mediation effect and/or by examining whether the mediation model differs across different groups of children (e.g. based on gestational age or neurodevelopmental conditions). These efforts would lead to better tailored intervention strategies for children born preterm.

To close, I applaud Ludyga et al. for their incredible efforts and hope this commentary will stimulate further discussions in this important topic.

## Data Availability

Not required.
